# Salinity-responsive histone PTMs identified in the gills and gonads of Mozambique tilapia (*Oreochromis mossambicus*)

**DOI:** 10.1186/s12864-024-10471-3

**Published:** 2024-06-11

**Authors:** Elizabeth A. Mojica, Yuhan Fu, Dietmar Kültz

**Affiliations:** Department of Animal Sciences & Genome Center, University of California - Davis, One Shields Ave., Meyer Hall, Davis, CA 95616 USA

**Keywords:** Epigenetics, Salinity stress, Euryhaline fish, Mass spectrometry, Stress-induced evolution

## Abstract

**Background:**

Histone post-translational modifications (PTMs) are epigenetic marks that can be induced by environmental stress and elicit heritable patterns of gene expression. To investigate this process in an ecological context, we characterized the influence of salinity stress on histone PTMs within the gills, kidney, and testes of Mozambique tilapia (*Oreochromis mossambicus*). A total of 221 histone PTMs were quantified in each tissue sample and compared between freshwater-adapted fish exposed to salinity treatments that varied in intensity and duration.

**Results:**

Four salinity-responsive histone PTMs were identified in this study. When freshwater-adapted fish were exposed to seawater for two hours, the relative abundance of H1K16ub significantly increased in the gills. Long-term salinity stress elicited changes in both the gills and testes. When freshwater-adapted fish were exposed to a pulse of severe salinity stress, where salinity gradually increased from freshwater to a maximum of 82.5 g/kg, the relative abundance of H1S1ac significantly decreased in the gills. Under the same conditions, the relative abundance of both H3K14ac and H3K18ub decreased significantly in the testes of Mozambique tilapia.

**Conclusions:**

This study demonstrates that salinity stress can alter histone PTMs in the gills and gonads of Mozambique tilapia, which, respectively, signify a potential for histone PTMs to be involved in salinity acclimation and adaptation in euryhaline fishes. These results thereby add to a growing body of evidence that epigenetic mechanisms may be involved in such processes.

**Supplementary Information:**

The online version contains supplementary material available at 10.1186/s12864-024-10471-3.

## Background

Epigenetic marks contribute to the regulation of gene expression patterns in the cells of eukaryotic organisms. Histone post-translational modifications (PTMs) and DNA methylation represent two classes of epigenetic marks, and they each can be influenced by numerous factors, including cell type, the organism’s developmental stage, and environmental conditions [1–5]. Under ideal circumstances, environmentally-induced epigenetic marks enable organisms to alter their gene expression in a way that better prepares them to survive and thrive within their environments. The evidence of this phenomenon occurring in nature is rapidly growing, particularly with the epigenetic mark of DNA methylation [6–8]. However, there is still a paucity of epigenetic research in fishes, especially in response to globally changing environmental factors, such as salinity [9].

The few studies conducted in this research area point to the involvement of DNA methylation in the acclimation and adaptation of fishes to salinity stress. The brown trout (*Salmo trutta*) presents an interesting example within this context [10]. Juveniles of this species can develop into either freshwater trout or migratory sea trout, which are genetically indistinguishable. Yet, the priority for conservation efforts has been to specifically enrich populations of the migratory morphotype, rather than the freshwater one. Following challenges in establishing the desired morphotype from hatchery-raised fish, it was found that feeding fish a high-salt diet altered the DNA methylation at key osmoregulatory genes, which led to an increased proportion of hatchery-raised fish that developed into migratory sea trout [10]. Examples of the putative involvement of epigenetic mechanisms in salinity adaptation come from studies on the three-spined stickleback (*Gasterosteus aculeatus*), which consist of several discrete populations locally adapted to different salinities. When compared between populations of sticklebacks adapted to different salinities, DNA methylation was found to vary at genes associated with osmoregulation [11, 12]. Moreover, once sticklebacks from a low salinity environment were acclimated to high salinity, they acquired intergenerationally stable patterns of DNA methylation that were similar to those found in the populations of sticklebacks locally adapted to high salinity [13].

Unlike DNA methylation, histone PTMs as an epigenetic mark have not yet been investigated in fishes experiencing salinity stress. Therefore, the purpose of this study was to characterize how histone PTMs respond to salinity stress in Mozambique tilapia (*Oreochromis mossambicus*). This species is strongly euryhaline, capable of tolerating salinities from freshwater (0 g/kg) to about four-times the salinity of seawater (120 g/kg), as long as fish have sufficient time to gradually acclimate to higher salinities [14, 15]. Time is needed during these acclimations so that the fish’s osmoregulatory organs (e.g., gills and kidney) can adjust their morphology and physiology in a way that switches their strategies for osmoregulation depending on environmental salinity [16]. To determine whether histone PTMs could be involved in this adjustment, and therefore salinity acclimation, we characterized the impact of salinity stress on histone PTMs in the gills and kidney. Furthermore, we tested whether salinity stress could impact histone PTMs in the testes, being representative of the male germ line, where epigenetic changes could be passed onto future generations. Such a process could facilitate salinity adaptation.

In this study, we imposed a variety of salinity treatments on Mozambique tilapia to test whether the intensity and duration of salinity stress differentially impacts histone PTMs in the gills, kidney, and testes. The first set of salinity treatments that we imposed on fish represented the strongest short-term salinity stress that Mozambique tilapia could tolerate. Due to their temporal limitations in salinity tolerance, Mozambique tilapia adapted to freshwater can only survive an immediate change in salinity up to 25 g/kg. However, they can temporarily tolerate an immediate change in salinity from freshwater to seawater (30 g/kg), as long as salinity decreases within six hours [17, 18]. Frequent changes between freshwater and seawater are regularly experienced in Mozambique tilapia when they inhabit tidal estuaries [19]. To mimic these large salinity changes, we exposed freshwater-adapted Mozambique tilapia to (1) freshwater, (2) seawater for two hours, or (3) seawater for two hours followed by a recovery in freshwater for two hours. The second set of salinity treatments was designed to reveal how long-term exposure to salinities near the upper tolerance limit of Mozambique tilapia influenced histone PTMs in different tissues. Additionally, it was designed to uncover whether the histone PTM response to severe salinity stress differed depending on the fish’s previous experience with salinity stress. Therefore, we exposed freshwater-adapted fish to (1) freshwater, (2) one “pulse” of severe salinity stress, where salinity gradually increased from freshwater to 82.5 g/kg, or (3) three pulses of severe salinity stress.

## Results

### Standards established for determining whether histone PTMs are salinity-responsive

**Fig. 1:**
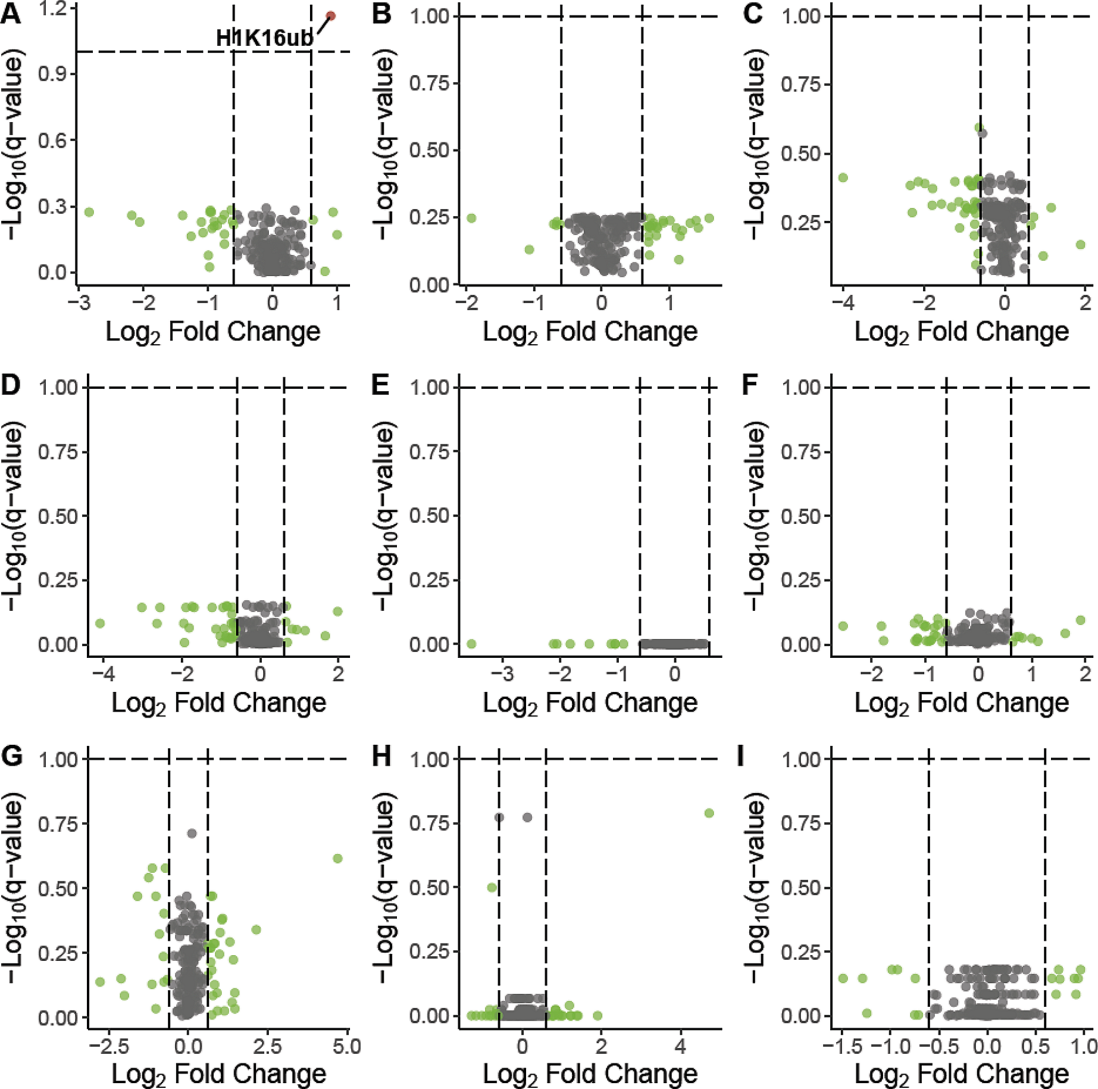
Impact of short-term salinity treatments on histone PTMs. Volcano plots depict the differences in histone PTMs between short-term salinity treatments. All histone PTMs were plotted based on their conditioned q-value and fold change. Panels A-C depict histone PTMs in the gills when comparisons were made between the fish exposed to SW and FW treatments (**A**), SW/FW and FW treatments (**B**), and SW and SW/FW treatments (**C**). Panels D-F depict histone PTMs in the kidney when comparisons were made between the fish exposed to SW and FW treatments (**D**), SW/FW and FW treatments (**E**), and SW and SW/FW treatments (**F**). Finally, panels G-I depict histone PTMs in the testes when comparisons were made between the fish exposed to SW and FW treatments (**G**), SW/FW and FW treatments (**H**), and SW and SW/FW treatments (I). Histone PTMs were colored according to their significance in terms of conditioned q-value (blue), fold change (green), both conditioned q-value and fold change (red), or neither (gray). The salinity-responsive histone PTM H1K16ub is labeled accordingly

**Fig. 2:**
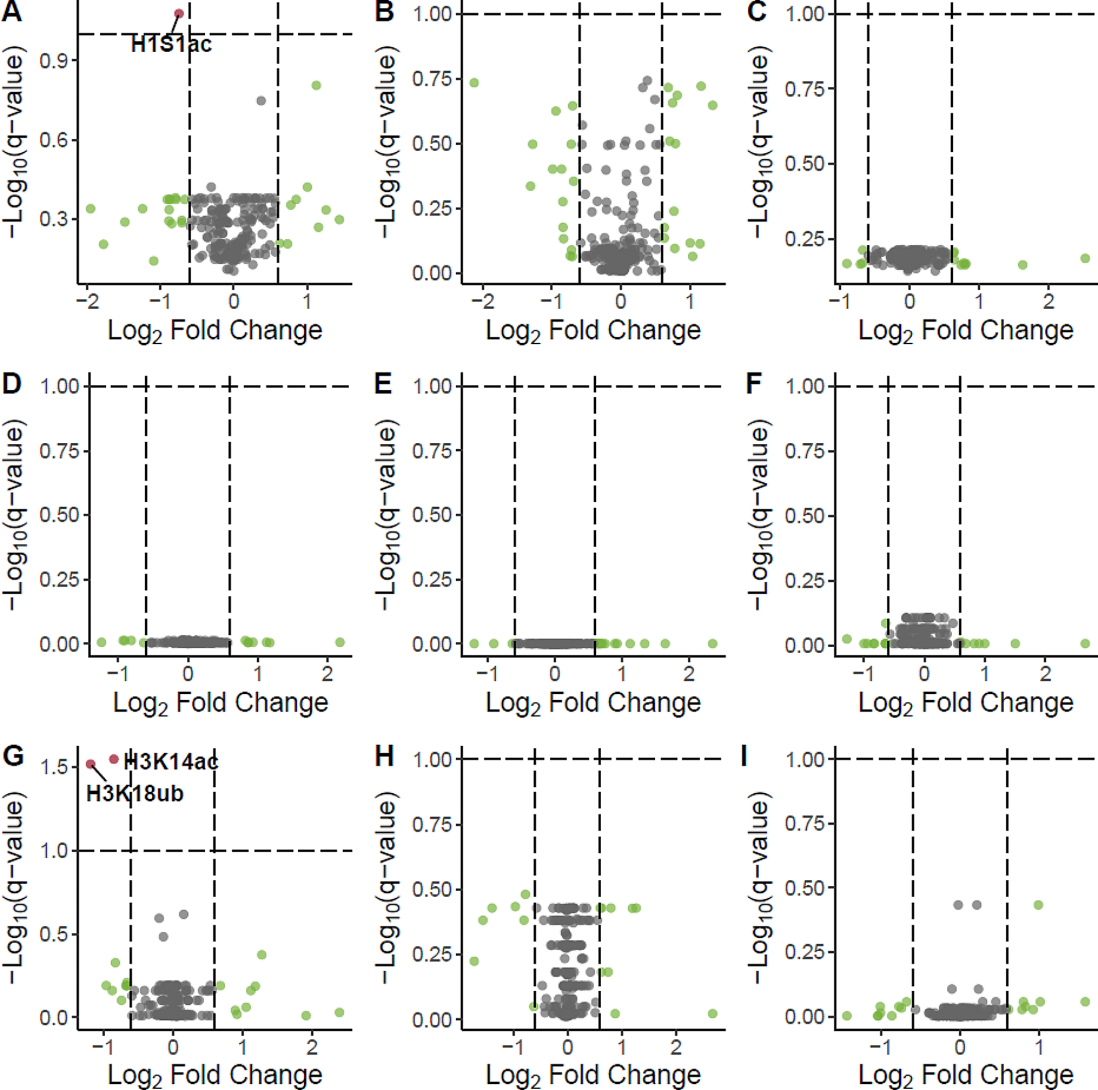
Impact of long-term salinity treatments on histone PTMs. Volcano plots depict the differences in histone PTMs between long-term salinity treatments. All histone PTMs were plotted based on their conditioned q-value and fold change. Panels A-C depict histone PTMs in the gills when comparisons were made between the fish exposed to S1 and S0 treatments (**A**), S3 and S0 treatments (**B**), and S3 and S1 treatments (**C**). Panels D-F depict histone PTMs in the kidney when comparisons were made between the fish exposed to S1 and S0 treatments (**D**), S3 and S0 treatments (**E**), and S3 and S1 treatments (**F**). Finally, panels G-I depict histone PTMs in the testes when comparisons were made between the fish exposed to S1 and S0 treatments (**G**), S3 and S0 treatments (**H**), and S3 and S1 treatments (**I**). Histone PTMs were colored according to their significance in terms of conditioned q-value (blue), fold change (green), both conditioned q-value and fold change (red), or neither (gray). Salinity-responsive histone PTMs are labeled according to their abbreviated names

For every sample of tissue collected from Mozambique tilapia following salinity treatments, a total of 3,504 peptides located on 25 different histone proteins were quantified. Values of histone peptide abundance were used to calculate the relative abundance, beta-value, and M-value of 221 biologically relevant histone PTMs using methods we have described in detail in a previous publication [20]. To determine whether histone PTMs were salinity-responsive, we compared the M-values for all 221 histone PTMs between the fish from different salinity treatments using t-tests. Because the salinity treatments were delivered as two experiments, we made comparisons between the fish from all short-term salinity treatments (Fig. 1), and, separately, we made comparisons between the fish from all long-term salinity treatments (Fig. 2). Comparisons were performed independently for each tissue analyzed.

Several histone PTMs were found to have a raw *p*-value < 0.05 when compared between fish from different salinity treatments; however, a correction was needed for determining the significance of these histone PTMs because multiple hypotheses (221) were tested within each comparison. We used Boca and Leek’s FDR regression method for multiple hypothesis testing correction because it provides high power by accounting for covariates [21, 22]. The covariate we chose for this correction was the type of modification (e.g., acetylation, methylation) of each histone PTM, as indicated by the Unimod accession number. The output of each test correction is a conditioned q-value, which represents the proportion of false discoveries in the list of significant results. We designated histone PTMs as salinity-responsive if they had a conditioned q-value of less than 0.1 when compared between fish exposed to different salinity treatments, as this indicates that less than 10% of all histone PTMs deemed significant are false discoveries. In this study, four histone PTMs met the specified criterion and were determined to be salinity-responsive in Mozambique tilapia. Two salinity-responsive histone PTMs were detected in the gills, with one histone PTM responding under short-term salinity stress and the other responding under long-term salinity stress. Another two histone PTMs were found to differ in the testes of fish exposed to long-term salinity stress. A complete account of how histone PTMs respond to salinity stress in the gills, kidney, and testes is presented in Supplemental Table 1.

### Short-term salinity stress altered one histone PTM in the gills

Freshwater-adapted Mozambique tilapia were given one of three short-term salinity treatments. Fish in the first treatment group (*SW*) were directly transferred from freshwater to seawater, then kept at seawater for exactly two hours before euthanasia. The fish from the second treatment group (*SW/FW*) were directly transferred from freshwater to seawater, kept at seawater for exactly two hours, then directly transferred back to freshwater. These fish were maintained in freshwater for an additional two hours as a recovery period before euthanasia. Fish in the control group (*FW*) were transferred from freshwater to another tank containing freshwater. When histone PTMs in the gills, kidney, and testes were compared between the fish from the three different short-term salinity treatments, one histone PTM met the criterion for being salinity-responsive (Fig. 1). This histone PTM was histone H1 isoform X1 lysine 16 ubiquitylation (H1K16ub), and it was found to be significantly different between the gills of fish exposed only to freshwater and the gills of fish exposed to seawater for two hours (*p*-value = 3.48e-04; conditioned q-value = 0.07).

**Fig. 3:**
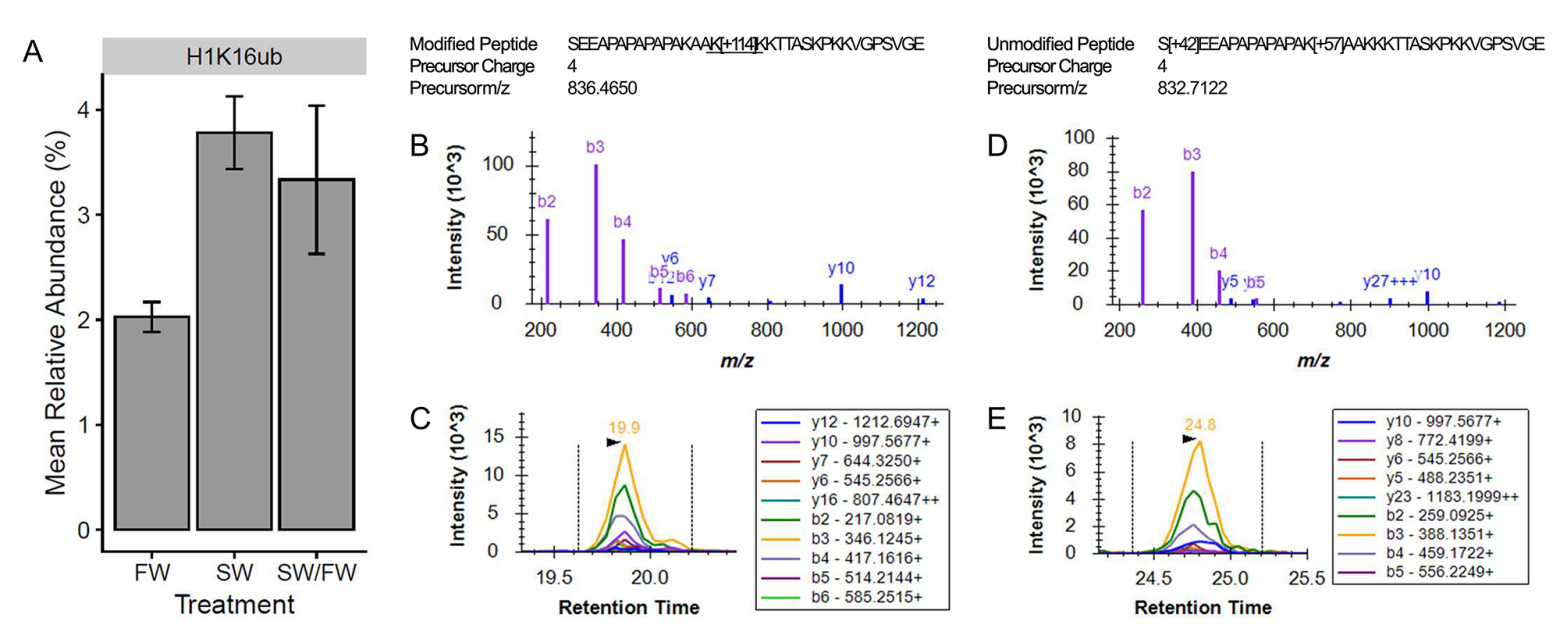
The influence of short-term salinity stress on H1K16ub. The mean relative abundance of H1K16ub in the gills is displayed for fish exposed to each of the short-term salinity treatments (**A**). Error bars represent the mean ± the standard error of the mean. The quantification of H1K16ub was based on the abundance of six modified versions of peptides and 39 unmodified versions of peptides. Panels B-C correspond to one of the modified peptides, SEEAPAPAPAPAKAAK[+ 114]KKTTASKPKKVGPSVGE, that contributed to H1K16ub quantification. The library spectrum (**B**) and an example peak (**C**) of this modified peptide are presented. Panels D-E depict a distinctive library spectrum (**D**) and example peak (**E**) from one of the unmodified peptides, S[+ 42]EEAPAPAPAPAK[+ 57]AAKKKTTASKPKKVGPSVGE

The influence of salinity stress on the global relative abundance of H1K16ub is displayed in Fig. 3. Short-term exposure to seawater increased relative abundance of this histone PTM compared to fish only exposed to freshwater. Fish only exposed to freshwater had an average relative abundance of H1K16ub of 2.03%. Exposure to seawater for two hours led to the significant increase in this histone PTM to a relative abundance of 3.78%. The relative abundance of H1K16ub in fish from the final treatment group, having been transferred from freshwater to seawater and back to freshwater, was not significantly different from either of the other treatment groups, as there was a higher variance in this histone PTM’s abundance. The mean relative abundance, however, remained high like it was in the fish exposed to seawater for two hours. In this case, the mean relative abundance of H1K16ub was 3.33%.

While H1K16ub was the only histone PTM to meet our criterion as a salinity-responsive histone PTM under short-term salinity stress, the volcano plots in Fig. 1 reveal further insight into how salinity stress influences histone PTMs in the gills, kidney, and testes. For example, salinity was shown to have a particularly low impact on histone PTMs in the kidneys. Some histone PTMs in the testes stood out from the rest for responding to salinity stress, although non-significantly. These included histone H1-like lysine 13 4-hydroxynonenalation, histone H2B.L4 lysine 75 dioxidation, and histone H1-like proline 11 dioxidation (see Supplemental Table 1).

### Long-term salinity stress altered three histone PTMs among the gills and gonads

Examinations into the effect of long-term salinity stress on histone PTMs in Mozambique tilapia consisted of three treatment groups. Fish in the first treatment group (*S1*) were exposed to one pulse of severe salinity stress, where salinity was gradually increased from freshwater (0 g/kg) to 82.5 g/kg, which is nearly three times the salinity of seawater. The second group of fish (*S3*) experienced the same pulse of severe salinity stress as the first treatment group, but instead of one pulse, they experienced three pulses of salinity stress over the course of 62 days. Fish in the final treatment group (*S0*) were handled as a control group and only ever experienced freshwater. The 221 histone PTMs quantified in this study were compared between fish from each group of long-term salinity treatments. These comparisons were performed separately for the gills, kidney, and testes (Fig. 2). Across these comparisons, three histone PTMs were identified as salinity responsive, and all significantly differed between the fish exposed only to freshwater and the fish exposed to one pulse of salinity stress.

**Fig. 4:**
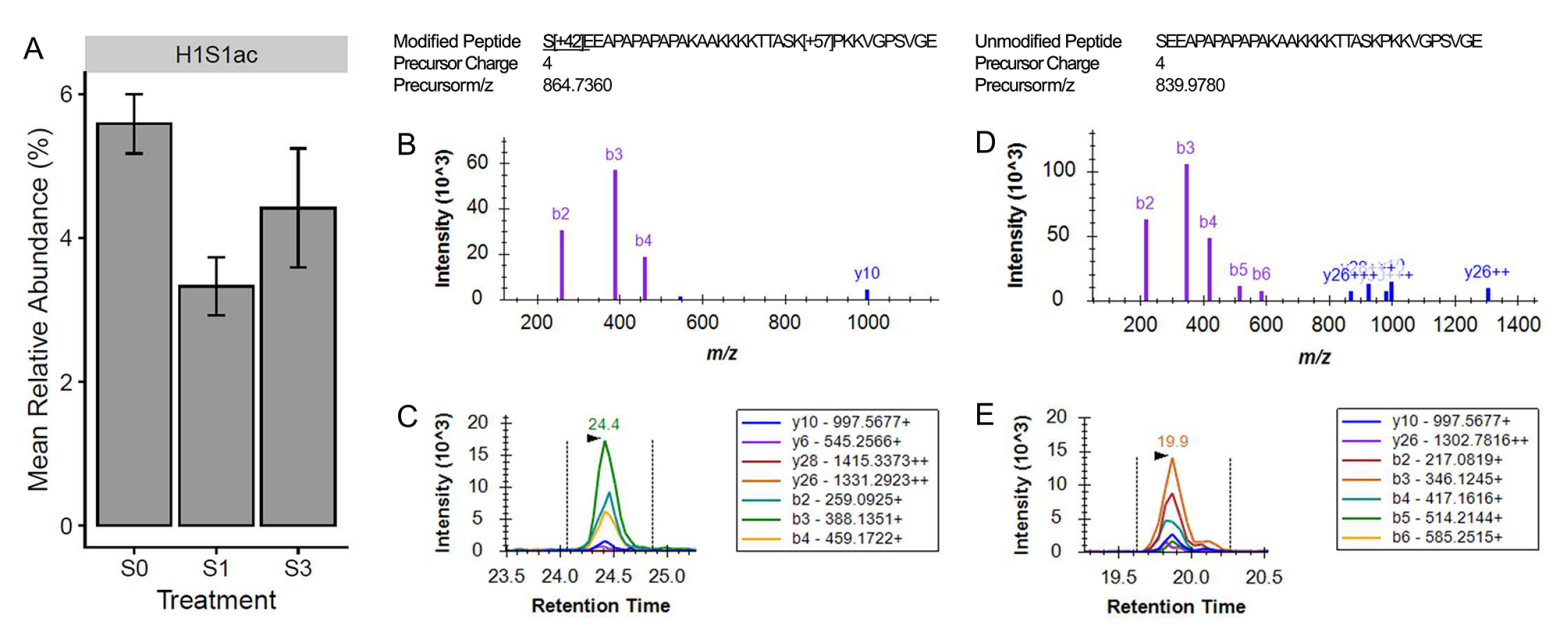
The influence of long-term salinity stress on H1S1ac. The mean relative abundance of H1S1ac in the gills is displayed for fish exposed to each of the long-term salinity treatments (**A**). Error bars represent the mean ± the standard error of the mean. The quantification of H1S1ac was based on the abundance of ten modified versions of peptides and 31 unmodified versions of peptides. Panels B-C represent one of the modified peptides that contributed to H1S1ac quantification, being S[+ 42]EEAPAPAPAPAKAAKKKKTTASK[+ 57]PKKVGPSVGE. For this modified peptide, the library spectrum (**B**) and an example peak (**C**) from the program Skyline are shown. Panels D-E correspond to one of the unmodified peptides, SEEAPAPAPAPAKAAKKKKTTASKPKKVGPSVGE, which has a distinctive library spectrum (**D**) and example peak (**E**)

**Fig. 5:**
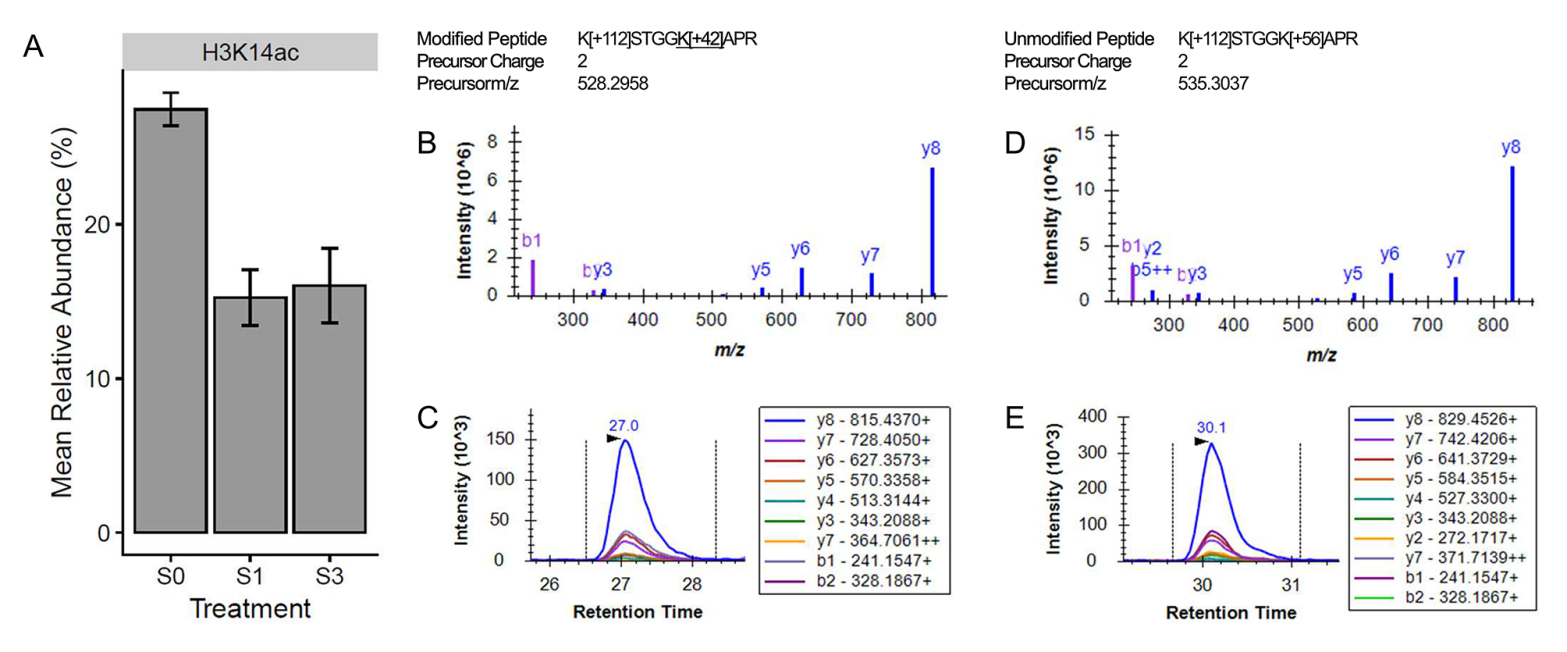
The influence of long-term salinity stress on H3K14ac. The mean relative abundance of H3K14ac in the testes is displayed for fish exposed to each of the long-term salinity treatments (**A**). Error bars represent the mean ± the standard error of the mean. The quantification of H3K14ac was based on the abundance of three modified versions of peptides and seven unmodified versions of peptides. Panels B-C represent one of the modified peptides that contributed to H3K14ac quantification, being K[+ 112]STGG**K[+ 42]**APR. For this modified peptide, the library spectrum (**B**) and an example peak (**C**) from the program Skyline are shown. Panels D-E correspond to one of the unmodified peptides, K[+ 112]STGGK[+ 56]APR, which has a distinctive library spectrum (**D**) and example peak (**E**)

**Fig. 6:**
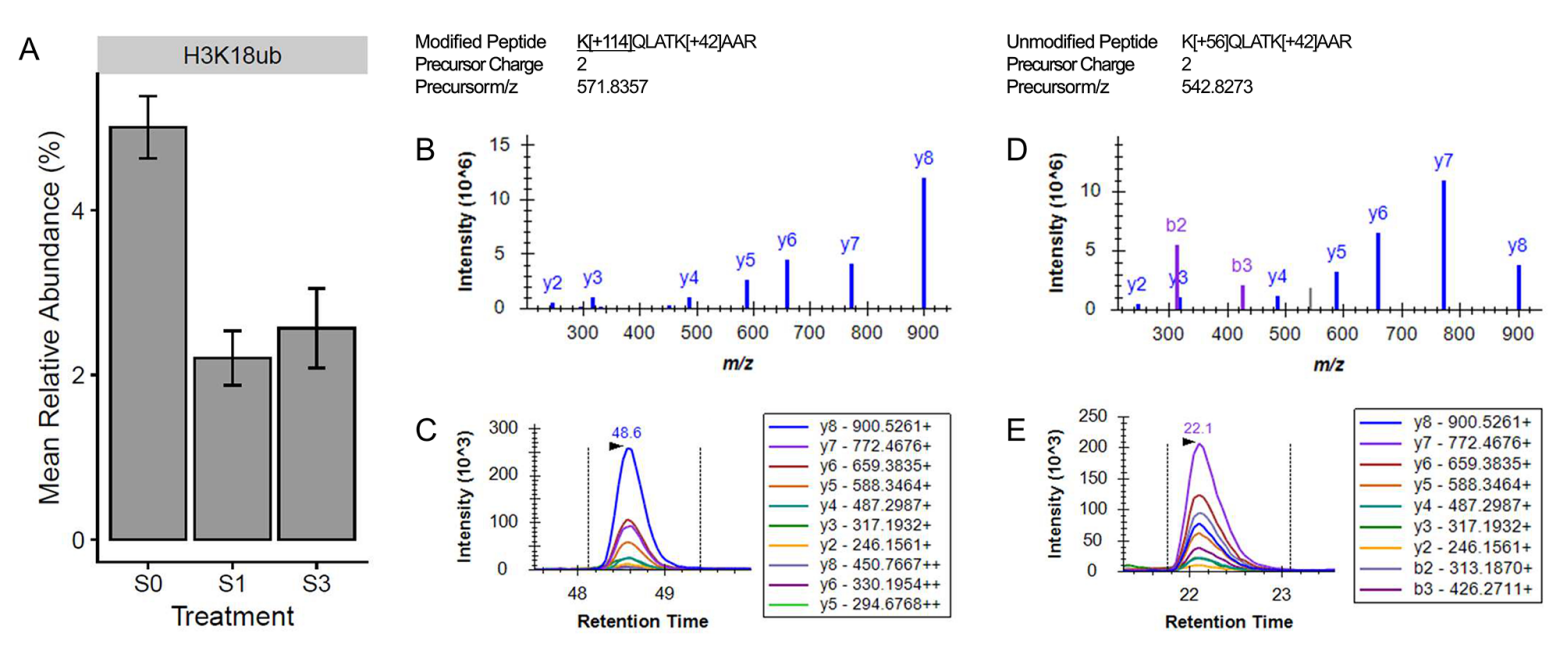
The influence of long-term salinity stress on H3K18ub. The mean relative abundance of H3K18ub in the testes is displayed for fish exposed to each of the long-term salinity treatments (**A**). Error bars represent the mean ± the standard error of the mean. The quantification of H3K18ub was based on the abundance of two modified versions of peptides and 13 unmodified versions of peptides. Panels B-C represent one of the modified peptides, K[+ 114]QLATK[+ 42]AAR, that contributed to H3K18ub quantification. The library spectrum (**B**) and an example peak (**C**) from the program Skyline are shown for this modified peptide. Panels D-E depict a distinctive library spectrum (**D**) and example peak (**E**) from one of the unmodified peptides, K[+ 56]QLATK[+ 42]AAR

In the gills, histone H1-like serine acetylation (H1S1ac; *p*-value = 5.23e-04; conditioned q-value = 0.08) was found to be significantly different between the two groups, with the global relative abundance being highest (5.59%) in the fish only exposed to freshwater and lowest (3.33%) in fish after exposure to one pulse of salinity stress (Fig. 4). In the testes, salinity stress led to the significant change in two histone PTMs: histone H3 lysine 18 ubiquitylation (H3K18ub; *p*-value = 2.68e-04; conditioned q-value = 0.03) and histone H3 lysine 14 acetylation (H3K14ac; *p*-value = 3.87e-04; conditioned q-value = 0.03). The manner in which salinity influenced the global relative abundance of these histone PTMs in the testes is depicted in Figs. 5 and 6. In the case of H3K14ac (Fig. 5), relative abundance was highest at 27.5% when fish were only ever exposed to freshwater. Once the fish were exposed to one pulse of severe salinity stress, the relative abundance decreased significantly to 15.3%. Similarly, fish exposed to three pulses of severe salinity stress exhibited a relative abundance of 16.0%; however, this value was not significantly different when compared to either of the other long-term salinity treatments. The case of H3K18ub (Fig. 6) is similar to that of H3K14ac. Relative abundance of H3K18ub was highest at 5.0% in the fish only ever exposed to freshwater. Upon exposure to one pulse of severe salinity stress, relative abundance decreased significantly to 2.2%. The relative abundance of H3K18ub remained low at 2.6% when fish were exposed to three pulses of salinity stress, but again this value was not significantly different from the other two long-term salinity treatments. It should be noted that no histone PTMs in the kidneys were found to respond significantly to salinity; rather, histone PTMs remained remarkably similar in the kidney, regardless of the salinity treatment experienced by the fish (Fig. 2).

### Human analogs of the salinity-responsive histone PTMs

To determine whether the salinity-responsive histone PTMs identified in this study are analogous to human histone PTMs, we performed sequence alignments between the primary protein structures of tilapia histone proteins and the corresponding human histone proteins (Supplemental Figs. 1–2) using Clustal Omega [23]. One sequence alignment was made between tilapia histone H1 isoform X1 (accession number XP_019210164.1), tilapia histone H1-like (accession number XP_019209845.1), and human histone H1 (accession number AAA63187.1). This alignment demonstrated that tilapia histone H1 isoform X1 lysine 16, on which we identified the salinity-responsive histone PTM of H1K16ub, aligned to human histone H1 arginine 24. Because ubiquitylation does not occur as a post-translational modification on arginine residues, we determined that there is no human analog of the salinity-responsive histone PTM of H1K16ub. This alignment also demonstrated that tilapia and humans share serine 1 on histone H1 isoforms, where acetylation was found to be salinity-responsive in the gills. A second sequence alignment was made between the histone H3 proteins in tilapia (accession number XP_005463512.2) and in humans (accession number AAN39284.1). The two salinity-responsive histone PTMs detected in tilapia, being H3K14ac and H3K18ub, aligned exactly to those in humans. Furthermore, these two histone PTMs have been previously detected and characterized across several species, including humans (Table 1).

**Table 1:** Salinity-responsive histone PTMs. For each salinity-responsive histone PTM, the full name, abbreviated name, and human analog are listed

Salinity-Responsive Histone PTM	Abbreviated Name of Histone PTM	Human Analog
H1 isoform X1 K16 ubiquitylation	H1K16ub	NA
H1-like S1 acetylation	H1S1ac	H1S1ac*
H3 K14 acetylation	H3K14ac	H3K14ac*
H3 K18 ubiquitylation	H3K18ub	H3K18ub*

* Previously detected in humans

## Discussion

### Four histone PTMs responded to salinity stress in Mozambique tilapia tissues

The goal of this study was to characterize the influence of salinity stress on histone PTMs in Mozambique tilapia. By measuring the histone PTM response to salinity of varying intensities and duration in the gills, kidney, and testes, we sought to determine whether histone PTMs could be involved in salinity acclimation and adaptation. This hypothesis was supported by the alteration of H1K16ub and H1S1ac in the gills, being an osmoregulatory organ, and by the alteration of H3K14ac and H3K18ub in the testes, being representative of the male germ line. Up until now, the investigation of salinity-responsive histone PTMs has been largely restricted to studies in plants [24–30]. Yet, despite the large taxonomic differences between fishes and plants, H3K14ac has now been shown to respond to salinity in tilapia in addition to soybean, tobacco, rice, and Arabidopsis [24, 25, 28, 30]. In plants, salinity stress influenced H3K14ac by causing its relative abundance to increase. The opposite effect was observed in tilapia. In this experiment, the fish only exposed to freshwater had the highest average relative abundance of H3K14ac in their testes at 27.5%. This value decreased significantly to 15.3% in fish exposed to one pulse of severe salinity stress, and it remained low at 16.0%, although non-significantly different, in the testes of fish exposed to three pulses of severe salinity stress.

H3K14ac is a highly studied histone PTM often associated with transcriptional activation [31–33], and it seems to exhibit dynamic regulation in response to stress, such that its abundance can decrease with stress [34], increase with stress [35], or decrease immediately after stress but later increase [36, 37]. This feature may explain why H3K14ac responded in opposite directions to salinity stress between our study in tilapia and studies conducted in plants. Moreover, a global significant increase in overall histone PTM abundance across the entire chromatin does not exclude the possibility of a significant reduction at specific genomic loci. Nonetheless, the decreased global relative abundance of H3K14ac observed in the testes of Mozambique tilapia is consistent with a recent study conducted on Nile tilapia (*Oreochromis niloticus*) [38]. When Nile tilapia were exposed to salinities near their upper tolerance limit, they experienced only subtle changes in spermatogenesis, but exhibited protein-level changes in the heat shock protein 70 (HSP70) and proliferating cell nuclear antigen (PCNA) in their testes [38]. As salinity increased, the abundance of HSP70 decreased, and the abundance of PCNA increased [38]. The abundance of HSP70 has previously been found to exhibit a positive correlation with H3K14ac [39, 40]. Therefore, a decrease in HSP70 would likely correspond to a decrease in the relative abundance of H3K14ac. Less expected was the trend observed for PCNA in the testes of Nile tilapia [38]. PCNA is used as a biomarker of cell proliferation and spermatogenesis [41], and its abundance has been positively correlated to that of H3K18ub, which we identified as another salinity-responsive histone PTM in Mozambique tilapia.

H3K18ub plays a critical role in maintaining patterns of DNA methylation after DNA replication [42, 43]. Like histone PTMs, DNA methylation can be impacted by an organism’s environment and contributes to upholding patterns of gene expression. Patterns of DNA methylation can endure across cycles of DNA replication, but due to the semi-conserved manner in which DNA replicates, DNA replication leaves DNA as hemi-methylated, such that the template strands of DNA are methylated in their “proper” pattern and the newly synthesized strands of DNA are unmethylated. Hemi-methylated DNA recruits the protein UHRF1 to begin the process of restoring proper DNA methylation patterns to newly synthesized DNA. UHRF1 ubiquitylates histone H3, with a preference for ubiquitylating H3K18 [42]. Following histone ubiquitylation, the DNA methylation enzyme DNMT1 is recruited to the genomic loci so that it can copy the DNA methylation pattern of the template strand onto the newly synthesized strand [43]. PCNA mediates the recruitment of DNMT1 to enhance the efficiency of DNA methylation restoration onto the newly synthesized strand of DNA [44, 45]. In this experiment, one pulse of severe salinity stress reduced the prevalence of H3K18ub in the testes of Mozambique tilapia. Although these results seem counterintuitive based on the previous study in Nile tilapia that found salinity stress to increase the abundance of PCNA in the testes [38], they are consistent with other findings that salinity stress can cause cell cycle arrest, including in *O. mossambicus* [46, 47].

The function of each salinity-responsive histone PTM identified in the gills of Mozambique tilapia, being H1K16ub and H1S1ac, is currently unknown. While H1S1ac has been previously detected in humans and mice, its function remained elusive in those contexts as well [48, 49]. H1K16ub does not have a human analog and has not been previously described. Ubiquitylation on histone H1 isoforms, however, has been associated with gene expression and the DNA damage response [50–52].

Histone PTMs are highly tissue-specific, and in a previous study, we found that the relative abundance of 91.59% of histone PTMs were significantly different between the gills, testes, and kidney of Mozambique tilapia [20]. Beyond those dramatic tissue differences, we show here that environmental salinity significantly influenced the relative abundance of two histone PTMs in the gills, two histone PTMs in the testes, and no histone PTMs in the kidney.

### The intensity and duration of the salinity challenge differentially impacted histone PTMs

The concept of salinity stress in fishes is highly complex. Even in the context of euryhaline fishes like Mozambique tilapia, which can tolerate a wide range of salinity, there are numerous caveats to this tolerance. How “stressful” a salinity challenge is to a euryhaline fish depends on several factors, including those specific to the environment, such as the starting salinity, the rate of salinity change, and the duration of altered salinity [17, 53]. Additionally, it includes factors specific to the fish, such as age and developmental stage, overall nourishment, and prior exposure to salinity stress [14, 54–56]. The histone PTM response to salinity in Mozambique tilapia tissues reflects the complex notion of salinity stress. In the gills, events of salinity stress led to changes in different histone PTMs depending on the event’s intensity and duration. In the testes, long-term salinity stress influenced two histone PTMs, but short-term salinity stress led to no significant response in histone PTMs.

The salinity treatments imposed on fish in this study were designed to investigate two additional features of the epigenetic response to salinity stress: (1) whether stress-induced histone PTMs persist after exposure to stress subsides, and (2) whether the histone PTM response to salinity stress depends on previous life experience. Investigating these features is especially relevant in Mozambique tilapia because many populations of this species inhabit hypersaline lakes, which regularly fluctuate in salinity depending on precipitation and evaporation [57, 58]. We therefore questioned whether the histone PTM response to salinity stress in fish would depend on the frequency of precipitation/evaporation cycles. Our hypothesis that stress-induced histone PTMs can persist after stress would have been supported if H1K16ub remained significantly higher in the gills of fish after fish were transferred back to freshwater from a two-hour exposure to seawater. We did not find sufficient evidence to support this hypothesis; however, as shown in Fig. 3, the average relative abundance of H1K16ub remained relatively high in the group of fish recovering from short-term exposure to seawater. Nonetheless, this value was not significantly different from either of the other salinity treatment groups due to increased variance in H1K16ub relative abundance. Our hypothesis that the histone PTM response to salinity stress depends on previous life experience would have been supported either if the salinity-responsive histone PTMs that changed with one pulse of severe salinity stress responded in opposite directions to fish experiencing three pulses of severe salinity stress, or if entirely different histone PTMs were affected by severe salinity stress when fish were exposed to either one pulse or three pulses of the stress. This hypothesis was not supported in this study. Although the histone PTMs of H3K14ac and H3K18ub were only found to be significantly different between the testes of fish exposed to one pulse of severe salinity stress and the testes of fish exposed to freshwater, fish exposed to three pulses of severe salinity stress exhibited a similar, though nonsignificant, response with these histone PTMs in the testes (Figs. 5 and 6).

### Limitations and recommendations for Future studies

Implicit to the experimental design used for this study was the considerable limitation that salinity-responsive histone PTMs could only be detected if they changed on a global, cellular level. Our study could not have captured how histone PTMs changed with salinity stress on a local, genomic loci-specific level. Given the functional role of histone PTMs in gene expression and maintenance, the genomic distribution of histone PTMs is likely to be of high importance. We anticipate that future studies would benefit from investigating the genomic distribution of the salinity-responsive histone PTMs identified here across various contexts of salinity exposures and time. Chromatin immunoprecipitation followed by sequencing (ChIP-seq) could be used for this purpose if appropriate antibodies for the identified histone PTMs are available [59]. Additionally, the histones surrounding genes of interest can be targeted for histone PTM analysis through methods of reverse-chromatin immunoprecipitation (R-ChIP), including Cas9 Locus-Associated Proteome (CLASP), Isolation of DNA Associated Proteins (IDAP), and Chromatin-of-Interest Fragment Isolation (CoIFI) [60, 61]. By targeting the histone PTMs associated with osmotically regulated genes, a more refined view of the histone PTM response to salinity could be obtained. Because four histone PTMs were detected for being salinity-responsive on a global level, their response across the genome must have been quite dramatic and consistent.

## Conclusions

In this study, Mozambique tilapia were exposed to salinity treatments that varied in intensity and duration before their gills, kidney, and testes were processed for histone PTM analysis. Of the 221 histone PTMs quantified and compared between tissues from fish in each salinity treatment, four were found to be salinity-responsive. Short-term salinity stress led to a significant increase in the relative abundance of H1K16ub in the gills, and long-term salinity stress led to the significant decrease in the relative abundances of H1S1ac in the gills and of both H3K14ac and H3K18ub in the testes. Notably, H3K14ac and H3K18ub have been well-documented in the scientific literature, and H3K14ac has been previously found to respond to salinity stress in plants. The results presented here complement a growing body of evidence that epigenetic mechanisms may be involved in the acclimation and adaptation of euryhaline fishes to salinity stress. We demonstrate that specific types of salinity stress can alter histone PTMs in an osmoregulatory organ, where stress-induced histone PTMs could contribute to salinity acclimation, and in the testes, where stress-induced histone PTMs have the potential to be meiotically inherited and thereby contribute to salinity adaptation. Future work will be needed to sufficiently characterize the nature of this histone PTM response to salinity stress and confirm any physiological or evolutionary function.

## Methods

### Salinity treatments and tissue Collection

In this study, a set of short-term salinity treatments and a set of long-term salinity treatments were delivered to a total of 42 adult Mozambique tilapia. The Mozambique tilapia were raised exclusively at the UC Davis Cole B facility and belonged to a lab-bred strain that originated from broodstock at the University of Stellenbosch, South Africa. The short-term and long-term salinity treatments were conducted as separate experiments, where 18 fish were used for the short-term salinity treatments, and 24 fish were used for the long-term salinity treatments. In each experiment, individual fish served as experimental units. A random number generator was used to assign treatment groups to each fish, based on the order in which the fish was collected from their initial tanks. All researchers were aware of the treatment group allocations at every stage of the experiments. For all treatments, salinity was adjusted using Instant Ocean (Instant Ocean, Cincinnati, OH, USA) and confirmed daily using a refractometer. Salinity was reported in units of g/kg, as this is the international standard, but it should be noted that values of salinity in units of g/kg are equivalent to those in the commonly used unit of ppt (i.e., 30 g/kg = 30 ppt). Fish were maintained on a 12 h light to 12 h dark schedule, and they were fed ad libitum daily.

For the short-term salinity treatments (Supplemental Fig. 3), each treatment group was composed of six fish as biological replicates, and fish were individually housed in 20 gallon tanks during their salinity exposures. Fish in the treatment group FW (handling control) were only ever exposed to freshwater (0 g/kg). In treatment group SW, fish were transferred directly from freshwater to seawater (30 g/kg) and kept there for exactly two hours. Finally, the fish in the treatment group SW/FW were transferred directly from freshwater to seawater, kept there for exactly two hours, then transferred directly back to freshwater and kept there for an additional two hours. Following exposures, all fish were euthanized and dissected for their gills, kidney, and in males, testes. A combination of pithing and cervical transection was used for euthanasia. Anesthetics were avoided in this process, with approval from the UC Davis Institutional Animal Care and Use Committee (IACUC), so that histone PTMs in the fish would not be altered by such an exposure. Due to the sexes of the fish randomly selected for this experiment, dissections yielded *n* = 6 gill samples, *n* = 6 kidney samples, and *n* = 3 testes samples per treatment group. Tissue samples were immediately placed in room temperature phosphate buffered saline (PBS) in preparation for histone PTM analysis, described below.

For the long-term salinity treatments (Supplemental Fig. 4), each treatment group was composed of eight fish, and fish were housed in 55 gallon tanks according to their treatment group. Fish in treatment group S0 acted as a control group and were only ever exposed to freshwater. Fish in treatment group S1 experienced a gradual shift in salinity from freshwater to a salinity of 82.5 g/kg, being nearly three times the salinity of seawater. For this treatment, salinity increased at a rate of 7.5 g/kg per day, then salinity was maintained at 82.5 g/kg for two days. This approximate rate of salinity change (6–8 g/kg per day) is commonly employed in studies on Mozambique tilapia, as it maximizes the upper salinity limit that freshwater-adapted tilapia can survive [15]. The physiological changes that Mozambique tilapia experience upon this gradual increase in salinity include extensive remodeling of gill epithelium, where mitochondria-rich ionocytes increase in number and size, and an increase in key compensatory proteins such as molecular chaperones, ion transporters, and proteins involved in the synthesis of compatible osmolytes [62–64]. In this study, fish in treatment group S3 were exposed to the same “pulse” of salinity stress as in treatment group S1, but instead of one pulse, they experienced three pulses of salinity stress. Therefore, salinity shifted from freshwater to 82.5 g/kg at a rate of 7.5 g/kg per day, then it decreased back to freshwater. The fish were maintained in freshwater for seven days before salinity was again increased to 82.5 g/kg. This pattern continued until three pulses of salinity stress were achieved after 62 days. For all long-term salinity treatments, salinity was increased by replacing 20% of the tank’s water volume with water containing a higher salinity. To control for the handling stress associated with water changes, 20% of the water volume was replaced in all tanks housing experimental fish whenever one treatment group experienced an increase in salinity. To decrease salinity following a pulse of salinity stress, 30% of the total volume of water was replaced with freshwater for four consecutive days. On the fifth day, 50% of the total water volume was replaced with freshwater, and on the sixth day, 100% of the water volume was replaced with freshwater. In order to change 100% of the water from each tank, fish were temporarily moved to a holding tank as water changes were made. As before, water changes were performed on all tanks housing experimental fish to control for handling stress. Fish were weighed following salinity treatments and found to have an average weight of 61 g. Subsequently, all fish were euthanized, and their gills, kidney, and testes (if male) were collected for histone PTM analysis. These dissections yielded *n* = 8 gill samples but only *n* = 6 kidney samples per treatment group, as two kidney samples were excluded from each treatment group due to low protein recovery. Dissections additionally yielded *n* = 6 testes samples per treatment group due to the sex of the fish randomly selected for this experiment. On average, the male fish had a gonadosomatic index of 0.35%. All tissue samples were immediately placed in room temperature PBS following dissection to begin histone PTM analysis as described below. Notably, the raw LCMS data files for these 24 fish were previously published without analyzing any effects of salinity when we thoroughly documented our methods for histone PTM analysis [20].

### Processing samples for histone PTM analysis

The workflow for histone PTM analysis was conducted as we have previously described [20]. As such, dissected tissues entered the workflow by being broken down into detached cells through a protocol of mechanical single cell suspension. Next, samples were enriched for histone proteins through histone acid extraction. Histone proteins were digested into peptides through the use of three parallel digestion methods: (1) using the protease trypsin (Promega Corporation, Madison, WI, USA) after chemical derivation of proteins through propionylation [65], (2) using the protease V8 (Thermo Scientific, Thermo Fisher Scientific, Inc., Rockford, IL, USA) in the buffer ammonium bicarbonate, and (3) using the protease V8 in the buffer sodium phosphate. Each digestion method produced a distinct set of histone peptides. Samples of histone peptides were then analyzed using liquid chromatography mass spectrometry using previously described parameters [66]. All DIA raw files were internally mass calibrated using DataAnalysis 4.1 (Bruker Daltonics) to yield a mean mass error of 0 ppm across all transitions. The absolute mass error allowed for any transition was 20 ppm, but the great majority had mass errors much smaller than 10 ppm (Supplemental Fig. 5). Previously constructed DIA assay libraries were used to quantify all tilapia-specific histone peptides. Values of histone peptide abundance (i.e., normalized area) were used to calculate the relative abundance, beta-value, and M-value of each histone PTM, as documented in detail before [20]. The relative abundance of each histone PTM was reported as an intuitive value that represents the percent of histones in a sample where a specific amino acid residue is occupied by the PTM of interest. To calculate this value from three sets of histone peptides, which corresponded to the three different digestion conditions, we separately used each set of histone peptides to divide the sum of the normalized area for all peptides containing the PTM of interest (e.g., H3K14ac) by the sum of the normalized area for all peptides containing the corresponding amino acid residue (e.g., H3K14), then multiply by 100. The three resulting values were averaged to equal the relative abundance of each histone PTM.

Similar to relative abundance, the beta-value represents the proportion, rather than percentage, of histones in a sample that contain the PTM of interest. A logit-transformation of the beta-value is called the M-value. The M-value of each histone PTM was calculated so that statistical analyses could be performed using values that follow a normal distribution as previously described [20]. In total, 221 biologically relevant histone PTMs were quantified in each sample using these methods.

### Statistical analyses

To test the effect of each salinity treatment on histone PTMs in Mozambique tilapia tissues, t-tests were performed on the M-values of all 221 quantified histone PTMs. Pairwise comparisons were made between fish exposed to each of the short-term salinity treatments, and these comparisons were made separately for the gills, kidney, and testes. Similarly, pairwise comparisons were made for each tissue between fish exposed to each of the long-term salinity treatments. The raw *p*-values resulting from each treatment comparison were corrected for multiple hypothesis testing. Boca and Leek’s FDR regression method was used for this purpose, as it provides a higher power than that of the commonly used Benjamini-Hochberg method by accounting for covariates [21, 22]. The covariate chosen for these tests was the type of modification (e.g., acetylation, methylation) of each histone PTM, and this was specified using the Unimod accession number. Statistical analyses were completed in the R programming environment (version 4.2.0) [67] using the R package *swfdr* [22]. Following analyses, multiple sequence alignments were performed using the program Clustal Omega [23] in order to determine the human analog of all salinity-responsive histone PTMs. Plots were prepared using the R packages *ggplot2* [68], *ggrepel* [69] and *tidyverse* [70] to visualize key results.

### Electronic supplementary material

Below is the link to the electronic supplementary material.

Supplementary Material 1

Supplementary Material 2

Supplementary Material 3

Supplementary Material 4

Supplementary Material 5

Supplementary Material 6

Supplementary Material 7

## Data Availability

All datasets generated and analyzed in this study are available at Panorama Public (https://panoramaweb.org/eam02kl.url, 10.6069/xdtd-4b83) and ProteomeXchange (PXD040557).

## Abbreviations

ac: Acetylation
ChIP-seq: Chromatin immunoprecipitation sequencing
CLASP: Cas9 Locus-Associated Proteome
CoIFI: Chromatin-of-Interest Fragment Isolation
FW: Freshwater
H1K16ub: Histone H1 isoform X1 lysine 16 ubiquitylation
H1S1ac: Histone H1-like serine 1 acetylation
H3K14ac: Histone H3 lysine 14 acetylation
H3K18ub: Histone H3 lysine 18 ubiquitylation
HSP70: Heat shock protein 70
IDAP: Isolation of DNA Associated Proteins
K: Lysine
PBS: Phosphate buffered saline
PCNA: Proliferating cell nuclear antigen
PTM: Post-translational modification
R-ChIP: Reverse-chromatin immunoprecipitation
S: Serine
SW: Seawater
ub: Ubiquitylation
