# Supplementary material for: Salinity-responsive histone PTMs identified in the gills and gonads of Mozambique tilapia (Oreochromis mossambicus)
Source: BMC Genomics. 2024 Jun 11;25:586. doi: 10.1186/s12864-024-10471-3 (PMC11167857; doi:10.1186/s12864-024-10471-3)
Supplement: Supplementary file 1 — Supplementary Material 1 [file 12864_2024_10471_MOESM1_ESM.docx]

**Supplemental Data Legends**

**Supplemental Table 1: Complete characterization of the histone PTM response to salinity stress in Mozambique tilapia.** For each combination of salinity treatment and tissue, the mean relative abundance of every histone PTM is presented. Furthermore, for every comparison of a histone PTM between salinity treatments, the log_2_ fold change, raw p-value, and conditioned q-value are provided.

**Supplemental Figure 1: Sequence alignment of tilapia and human histone H1 proteins.**

Clustal Omega was used to align the amino acid sequence of tilapia histone H1 isoform X1 (accession number XP_019210164.1), tilapia histone H1-like (accession number XP_019209845.1), and human histone H1 (accession number AAA63187.1).

**Supplemental Figure 2: Sequence alignment of tilapia and human histone H3 proteins.** Clustal Omega was used to align the amino acid sequence of tilapia histone H3 (accession number XP_005463512.2) and human histone H3 (accession number AAN39284.1).

**Supplemental Figure 3: Experimental design for testing effects of short-term salinity stress.** Three groups of six fish each received a salinity treatment designed to elicit large differences in plasma osmolality across treatment groups. The fish from treatment group FW acted as a control and were only ever exposed to freshwater. The fish from treatment group SW experienced a direct transfer from freshwater to seawater and were kept there for two hours before dissection. Finally, the fish from treatment group SW/FW experienced a direct transfer from freshwater to seawater, were kept in seawater for two hours, then were transferred back to freshwater and kept there an additional two hours before being dissected. Red points indicate the time at which fish were dissected.

**Supplemental Figure 4: Experimental design for testing effects of long-term salinity stress.** Three groups of eight fish were each exposed to a different salinity treatment over the course of 62 days. Fish from treatment group S0 were only ever exposed to freshwater. Fish from treatment group S1 were maintained in freshwater before experiencing one pulse of severe salinity stress, which was delivered gradually and reached a maximum salinity of 82.5 g/kg. Fish from treatment group S3 experienced three pulses of severe salinity stress before their dissection. Red points indicate time of dissection, and blue dashed boxes indicate each pulse of severe salinity stress.

**Supplemental Figure 5: Mass Error Histogram.** The mass error histogram is presented for each Skyline file individually processed in this study. Panels A-C correspond to the files containing gill samples from fish exposed to short-term salinity treatments, where tissues were processed using V8 in ammonium bicarbonate (A), V8 in sodium phosphate (B), and trypsin (C). Panels D-F correspond to the files containing gill samples from fish exposed to long-term salinity treatments, where tissues were processed using V8 in ammonium bicarbonate (D), V8 in sodium phosphate (E), and trypsin (F). Panels G-I correspond to the files containing kidney samples from fish exposed to short-term salinity treatments, where tissues were processed using V8 in ammonium bicarbonate (G), V8 in sodium phosphate (H), and trypsin (I). Panels J-L correspond to the files containing kidney samples from fish exposed to long-term salinity treatments, where tissues were processed using V8 in ammonium bicarbonate (J), V8 in sodium phosphate (K), and trypsin (L). Panels M-O correspond to the files containing testes samples from fish exposed to short-term salinity treatments, where tissues were processed using V8 in ammonium bicarbonate (M), V8 in sodium phosphate (N), and trypsin (O). Panels P-R correspond to the files containing testes samples from fish exposed to long-term salinity treatments, where tissues were processed using V8 in ammonium bicarbonate (P), V8 in sodium phosphate (Q), and trypsin (R).
