# Supplementary material for: Salinity-responsive histone PTMs identified in the gills and gonads of Mozambique tilapia (Oreochromis mossambicus)
Source: BMC Genomics. 2024 Jun 11;25:586. doi: 10.1186/s12864-024-10471-3 (PMC11167857; doi:10.1186/s12864-024-10471-3)
Supplement: Supplementary file 2 — Supplementary Material 2 [file 12864_2024_10471_MOESM2_ESM.pdf]

CLUSTAL O(1.2.4) multiple sequence alignment

```
AAA63187.1          MSETAPAAPAAPAPAEKTPVKKKARKSAGAAKRKASGPPVSELITKAVAASKERSGVSLA        60
XP_019210164.1      MSEEAPAPAPAPA-------KAAK-KKTTASKPKKVGPSVGELIVKAVAASKERSGVSAA        52
XP_019209845.1      MSEEAPAPAPAPA-------KAAKKKKTTASKPKKVGPSVGELIVKAVAASKERSGVSTA        53
                    *** ***   ***       *    *.: *:* *  ** *.***.************* *

AAA63187.1          ALKKALAAAGYDVEKNNSRIKLGLKSLVSKGTLVQTKGTGASGSFKLNKKAASGEAKPKA        120
XP_019210164.1      ALKKALAAGGYDVDKNKARVKTAIKSLVAKGTLVQTKGTGASGSFKMNKKATESKAKKPA        112
XP_019209845.1      ALKKALAAGGYDVDKNKARVKTAIKSLVAKGTLVQTKGTGASGSFKMNKKATESKAKKPA        113
                    ********.****:**::*:* .:****:*****************:****:..:**  *

AAA63187.1          K-------KAGAAKAKKPAGAAKKPKKATGAATPKKSAKKTPKKAKKPAAAAGAKKAKSP        173
XP_019210164.1      KKAAPKAKKPAAAKAKKPAAAAKKSPKKAAAAKKPAAAKKSPKKAKKPAAAAKK-VTKSP        171
XP_019209845.1      KKAAPKAKKPAAAKAKKPAAAAKKSPKKAAAAKKPAAAKKSPKKAKKPAAA-KK-APKSP        171
                    *       * .********.****  * :.**.   :***:**********      ***

AAA63187.1          KKAKAAKPKKAPKSPAKAKAVKPKAAK---PKTAKPKAAKPKKAAAKKK-----      219
XP_019210164.1      K--------KAAKSPKKVLKKAPAAKKSPAKKAAKPKVKKAAT-AAKKKCLRFI      216
XP_019209845.1      K--------KAAKSPKKVLKKAPAAKKSPAKKAAKPKVKKAAT-AAKKK-----      211
                    *        ** *** *.    * * *    *:****. *  . *****
```

Alt+Q
