# Supplementary material for: Salinity-responsive histone PTMs identified in the gills and gonads of Mozambique tilapia (Oreochromis mossambicus)
Source: BMC Genomics. 2024 Jun 11;25:586. doi: 10.1186/s12864-024-10471-3 (PMC11167857; doi:10.1186/s12864-024-10471-3)
Supplement: Supplementary file 3 — Supplementary Material 3 [file 12864_2024_10471_MOESM3_ESM.pdf]

```
CLUSTAL O(1.2.4) multiple sequence alignment


XP_005463512.2      MARTKQTARKSTGGKAPRKQLATKAARKSAPATGGVKKPHRYRPGTVALREIRRYQKSTE        60
AAN39284.1          MARTKQTARKSTGGKAPRKQLATKVARKSAPATGGVKKPHRYRPGTVALREIRRYQKSTE        60
                    ************************.***********************************

XP_005463512.2      LLIRKLPFQRLVREIAQDFKTDLRFQSSAVMALQEASEAYLVGLFEDTNLCAIHAKRVTI        120
AAN39284.1          LLIRKLPFQRLMREIAQDFKTDLRFQSSAVMALQEACESYLVGLFEDTNLCVIHAKRVTI        120
                    ***********:************************.*:************.********

XP_005463512.2      MPKDIQLARRIRGRGLKLI 139
AAN39284.1          MPKDIQLARRIRGERA--- 136
                    *************.
```
