# Supplementary figures and images for: Salinity-responsive histone PTMs identified in the gills and gonads of Mozambique tilapia (Oreochromis mossambicus)
Source: BMC Genomics. 2024 Jun 11;25:586. doi: 10.1186/s12864-024-10471-3 (PMC11167857; doi:10.1186/s12864-024-10471-3)

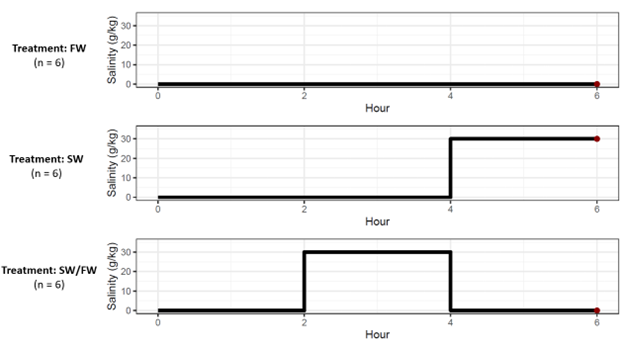

Supplement: Supplementary file 4 — Supplementary Material 4 [file 12864_2024_10471_MOESM4_ESM.png]

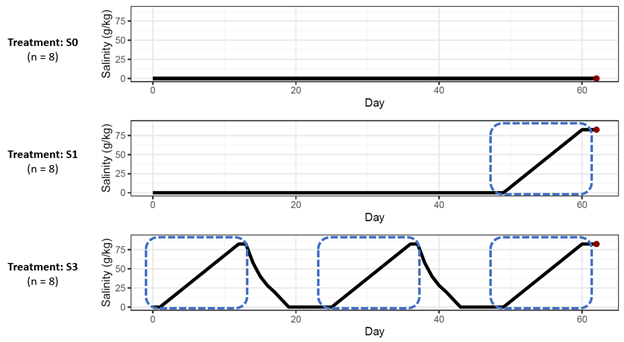

Supplement: Supplementary file 5 — Supplementary Material 5 [file 12864_2024_10471_MOESM5_ESM.png]

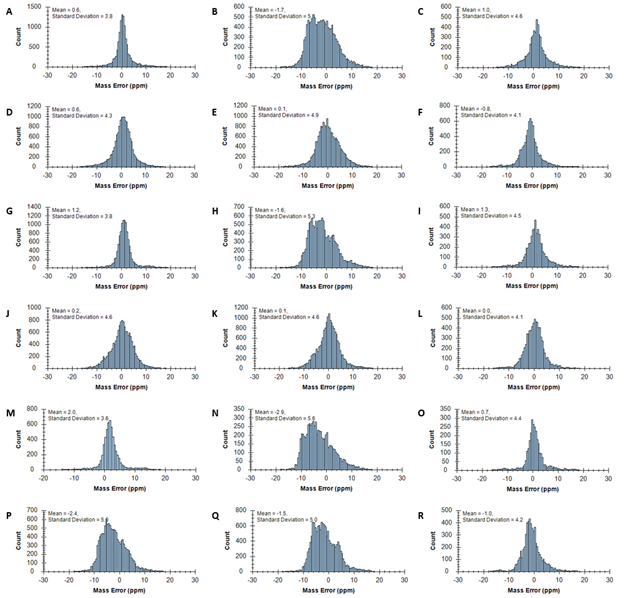

Supplement: Supplementary file 6 — Supplementary Material 6 [file 12864_2024_10471_MOESM6_ESM.png]
